# Ebola Virus Neutralizing Antibodies Detectable in Survivors of theYambuku, Zaire Outbreak 40 Years after Infection

**DOI:** 10.1093/infdis/jix584

**Published:** 2017-12-14

**Authors:** Anne W Rimoin, Kai Lu, Matthew S Bramble, Imke Steffen, Reena H Doshi, Nicole A Hoff, Patrick Mukadi, Bradly P Nicholson, Vivian H Alfonso, Gerrard Olinger, Cyrus Sinai, Lauren K Yamamoto, Christina M Ramirez, Emile Okitolonda Wemakoy, Benoit Kebela Illunga, James Pettitt, James Logue, Richard S Bennett, Peter Jahrling, David L Heymann, Peter Piot, Jean Jacques Muyembe-Tamfum, Lisa E Hensley, Graham Simmons

**Affiliations:** 1 Department of Epidemiology, Jonathan and Karin Fielding School of Public Health, University of California, Los Angeles; 2 Blood Systems Research Institute, and Department of Laboratory Medicine, University of California, San Francisco; 3 Department of Genetic Medicine Research, Children’s Research Institute, Children’s National Medical Center, Washington, District of Columbia; 4 Institut National de Recherche Biomedicale, Kinshasa, DRC; 5 Institute for Medical Research, Durham Veterans Affairs Medical Center, North Carolina; 6 Integrated Research Facility at Fort Detrick; 7 Emerging Viral Pathogens Section, National Institute of Allergy and Infectious Diseases, National Institutes of Health, Frederick, Maryland; 8 Department of Biostatistics, Jonathan and Karin Fielding School of Public Health, University of California, Los Angeles; 9 Kinshasa School of Public Health, Kinshasa; 10 Direction de la Lutte Contre les Maladies, Ministère de la Sante, DRC; 11 Chatham House Center on Global Health Security, London, UK; 12 London School of Hygiene and Tropical Medicine, London, UK

**Keywords:** Ebola virus, Democratic Republic of the Congo, virus neutralization, serological response, filovirus

## Abstract

The first reported outbreak of Ebola virus disease occurred in 1976 in Yambuku, Democratic Republic of Congo. Antibody responses in survivors 11 years after infection have been documented. However, this report is the first characterization of anti-Ebola virus antibody persistence and neutralization capacity 40 years after infection. Using ELISAs we measured survivor’s immunological response to Ebola virus Zaire (EBOV) glycoprotein and nucleoprotein, and assessed VP40 reactivity. Neutralization of EBOV was measured using a pseudovirus approach and plaque reduction neutralization test with live EBOV. Some survivors from the original EBOV outbreak still harbor antibodies against all 3 measures. Interestingly, a subset of these survivors’ serum antibodies could still neutralize live virus 40 years postinitial infection. These data provide the longest documentation of both anti-Ebola serological response and neutralization capacity within any survivor cohort, extending the known duration of response from 11 years postinfection to at least 40 years after symptomatic infection.

## Teaser

Duration of immunity against Ebola virus among survivors remains unclear. We assessed serological immune profiles and retention of Ebola virus neutralizing antibodies in 14 survivors of the 1976 Yambuku outbreak 40 years postinfection, providing the longest documentation of such measures reported.

*Ebolavirus* Zaire, abbreviated herein as EBOV, a member of the *Filoviridae* family, is a highly virulent pathogen that is often associated with high mortality rates in humans [1]. Outbreaks have occurred with increasing frequency throughout Central and Western Africa, with mortality rates ranging from 25% to 90% [2–5]. EBOV is spread via direct or indirect transmission through contact with bodily fluids such as vomit, blood, and diarrhea. Transmission can be halted with early diagnosis, effective surveillance with contact tracing, patient isolation, and safe burial practices [5]. However, the failure of quarantine methods in the recent West African outbreak have highlighted the fragility of health infrastructures and the urgency to better understand the ecology of EBOV and develop targeted and effective viral therapies [6].

**Figure 1.**
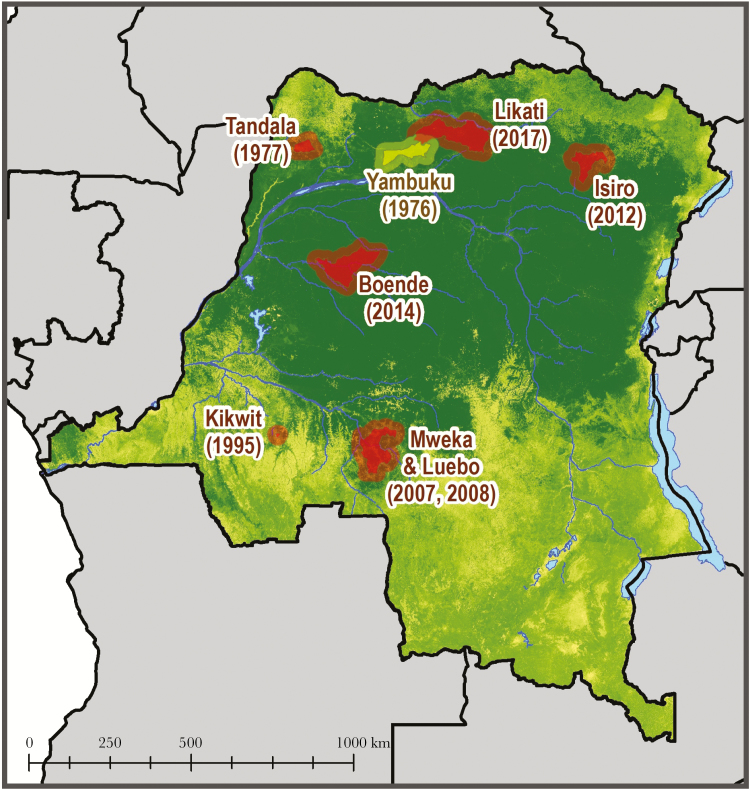
Map highlighting the location and year of prior Ebola virus Zaire outbreaks within the Democratic Republic of the Congo.

The first documented outbreak of Ebola virus disease (EVD) caused by EBOV occurred in 1976 in the northwestern part of the Democratic Republic of Congo (DRC, formerly Zaire) [2, 7]. The index case was a 44-year-old man who was treated at Yambuku hospital for epistaxis, dysentery, and fever. Between August 26 and November 5, 318 cases were recorded, resulting in 280 deaths, and 38 serologically confirmed survivors, with a case-fatality rate of 88%. The factors associated with transmission included receipt of injection with a reusable syringe, and close contact with an acute EVD case [2]. Six additional outbreaks have since occurred in DRC between 1977 and 2014 [8], and in May of 2017 there was yet another Ebola outbreak that occurred within the Likati Health Zone, a region just 150 km away from where the 1976 outbreak occurred (Figure 1).

The duration of immunity against EBOV among survivors still remains unclear, although Natesan et al [9] (Ebola virus Sudan, Ebola virus Bundibugyo, and Marburg virus) and Corti et al [10] (Ebola virus Zaire, Kikwit outbreak) have both demonstrated that antibodies specific to *Filoviridae* are maintained in survivors up to 11 years postinfection. The duration of antibody response and neutralization potentials in survivors who were infected more than 11 years prior is unknown. While documentation of long-term antibody maintenance in Ebola survivors remains limited, others have demonstrated that antibody presence against other viruses such as Lassa can be retained for significant durations of time [11]. Furthermore, no retrospective characterization of the immunological responses in survivors of the 1976 Yambuku outbreak has been previously reported. To investigate this, we obtained blood samples from 14 remaining survivors infected during the 1976 Yambuku outbreak, to assess the serological immune profile and retention of EBOV neutralizing antibodies 40 years postinfection, providing the longest documentation of such measures in EVD cases.

## METHODS

In January 2016, 14 survivors from the 1976 Yambuku EVD outbreak were identified using DRC Ministry of Health reports. Six of the identified participants were considered confirmed cases and enrolled in the plasmapheresis study conducted after 1976, with symptoms and molecular analyses that were considered positive at the time [12]. The remaining 8 participants were suspected cases based on Ministry of Health reports along with in-person interviews, and confirmation from health-care workers present during the outbreak. Participants were asked to complete a detailed questionnaire, which included demographic characteristics, health history, current health status, and exposure to wildlife and potentially infected humans. Upon review of the questionnaire responses, all 14 survivors reported symptoms of EVD (fever or unexplained bleeding or any three of the following: headache, myalgia, rash, vomiting, diarrhea, hiccups, breathing problems, or difficulty swallowing [13]) during the outbreak between August and November 1976. Study participants were also asked to complete a physical assessment, which included weight, height, and blood pressure. Blood specimens were obtained from participants by venipuncture in vacutainer tubes (BD Biosciences). After processing, aliquots of serum, plasma and buffy coat were frozen and stored in a liquid nitrogen dry shipper at the Institut National de Recherche Biomedicale in Kinshasa and shipped to collaborating institutions.

### Enzyme-linked Immunosorbent Assays

Human anti-EBOV Zaire Glycoprotein (GP) IgG titers were measured using a commercially available enzyme-linked immunosorbent assay (ELISA) kit (Alpha Diagnostic International, Inc.) following the manufacture’s protocol. Optical density values at 450 nm (OD_450_) were recorded for each sample at a 1:250 dilution in duplicate. A sample was determined mildly reactive if the serum antibody concentration was greater than 1.0 Units/mL of the purified IgG control calibrator. The sample was considered strongly reactive if they harbored titers greater than 5 Units/mL, a more conservative cutoff than what was used in previous reports, where a 4.7 U/mL cutoff yielded 96.7% sensitivity and 97.7% specificity [14].

Human anti-EBOV Zaire nucleoprotein (NP) titers were measured using commercially available ELISA kits (Alpha Diagnostics International) and were performed in San Francisco, CA. The assays were performed following the manufacturer’s instructions at a 1:200 sample dilution in duplicate and the absorbance was read at 450 nm. A sample was determined mildly reactive if the serum antibody concentration was greater than the 1.0 Units/mL control calibrator and strongly reactive if they harbored titers greater than 5 Units/mL.

### Luciferase Immunoprecipitation System

The C-terminal domain of Mayinga EBOV Zaire viral protein 40 (VP40; bp 583–981) was cloned into the pRen2 plasmid and transfected into Cos-1 cells generating *Renilla* luciferase antigen fusion proteins. Cell lysates were harvested and used in immunoprecipitation assays with Protein A/G conjugated agarose beads as described by Burbelo et al [15]. In the final step, the beads were washed 4 times with buffer A and 1 time with phosphate buffered saline using a vacuum manifold before measuring luciferase activities using the *Renilla* luciferase assay system substrate (Promega). VP40 positivity was determined if the relative luciferase signal postimmunoprecipitation was at least 3 standard deviations greater than the background signal, as determined from an average of 8 previously identified negative serum samples. Positive controls were obtained from human convalescent patient sera collected during the 2014 Boende, DRC, EBOV outbreak [16]. Data are presented as relative light units (RLU) of patient over RLU of negative controls; values greater than 1, are considered positive.

### Pseudotype Virus Neutralization Assay

Pseudotype viruses were generated as described previously [17] by using 25 μg Mayinga Zaire Ebola virus glycoprotein (EBOV-GP) [18] or, as a negative control, vesicular stomatitis virus glycoprotein (VSV-G) [19] expression plasmids, respectively, and 10 μg pNL-Luc viral backbone obtained through the NIH AIDS Reagent Program, Division of AIDS, NIAID, NIH (catalog #3418) [20]. The pseudotypes contained in the culture supernatant were pelleted through a 20% sucrose cushion by ultracentrifugation for 1.5 hours at 4°C and 27000 rpm in a SW-28 rotor and the viral pellet resuspended in 1/100 volume of Hank’s buffered saline solution (HBSS) overnight at 4°C. The pseudotype virus stocks were aliquoted and stored at −80°C. Pseudotype viruses were titered on human rhabdomyosarcoma (RD) cells to determine the volume required to yield 10^4^ RLU luciferase activity in cell lysates 48 hours postinfection. Two pseudotype virus neutralization assays were performed as previously described [21]. Pseudotype virus was incubated with serial dilutions of sera (1:50, 1:250, 1:1250, 1:6250) before infection of RD cells. All infections were completed in duplicate wells and each plate contained identical controls, including uninfected cells, cells infected in absence of serum, and cells infected in presence of negative control serum (US donor) or positive control serum from a recent, confirmed Ebola survivor. Infection rates in presence of human serum samples were expressed as percentage of infection in presence of negative control serum. To determine neutralizing capability, serum from the patients had to at least neutralize approximately 50% of virus compared to appropriate control.

### EBOV-like Particle Entry Inhibition Assay

EBOV Zaire-like particles (VLPs) produced using VP40-GLucN were prepared as previously described [22] and used in a G-Luciferase (GLuc) complementation assay for entry inhibition. VLPs were incubated with serial dilutions of sera (1:50, 1:250, 1:1250, 1:6250) before spin-infection of target cells (RD cells transiently expressing GLucC), in duplicate. GLuc activity in cell lysates was measured using a commercial kit (Pierce) according to the manufacturer’s instructions and expressed as percentage of luciferase activity in the presence of negative control serum.

### Plaque Reduction Neutralizing Test 50% and 80%

The plaque reduction neutralization test (PRNT50 or 80) is a neutralization assay in which live virus is incubated in the presence of serial dilutions of test samples under BSL-4 containment conditions. The virus/sample mixture is then incubated with cells, and nonneutralized virus infects cell monolayers. The antibody titer can be calculated by comparing the number of virus plaques in each sample dilution well to the virus-only control wells (no test sample). Samples were serial diluted (1:2) in Dulbecco’s Modified Eagle’s Medium (DMEM) over a range of 1:30 to 1:10240, mixed at a 1:1:1 ratio with the C05 isolate of Ebola Makona and either complement (Cedar Lane Labs standard guinea pig complement) or media and incubated at 37°C with 5% CO_2_ for 1 hour. Following incubation, 300 µL of virus/serum inoculum was added to triplicate wells of a 6-well plate containing 90%–100% confluent Vero E6 cells and rocked every 15 minutes for 1 hour. Following incubation, 2 mL of overlay (1.25% Avicel in minimal essential media) was added to each well before incubation at 37°C with 5% CO_2_ for 8 days. After incubation, the overlay was removed, and the cell monolayers fixed for 30 minutes at room temperature with 1 mL of a 0.2% crystal violet solution in 10% neutral-buffered formalin. The plates were washed with tap water and dried, and the total number of plaque forming units (PFUs) were counted and recorded for each well. An anti-Ebola virus antibody from IBT Bioservices (#01-0004) was used as a positive control for each assay. The PRNT50 was calculated by identifying the first dilution with 50% or 80% fewer plaques compared to the average number of plaques observed in virus-only wells.

### Cells

Human embryonic kidney 293T, human muscle RD, and African green monkey COS-1 cells were grown in Dulbecco’s modified essential medium (Hyclone) supplemented with 10% fetal bovine serum, l-glutamine, nonessential amino acids, and antibiotics penicillin and streptomycin (all Gibco) and incubated at 37°C with 5% CO_2_. For PRNT50 assays: African green monkey kidney cells (VERO C1008 (E6), ATCC, CRL-1586) were maintained in DMEM high glucose with l-glutamine (Lonza 12-604Q) containing 10% heat-inactivated fetal bovine serum (Sigma Life Sciences, F2442). The cells were seeded one day prior at 4 × 10^4^ cells/well in 96-well plates (Greiner bio-one 655948) and 1 × 106 cells/well in 6-well plates (Corning 3506), respectively, and incubated at 37°C with 5% CO_2_.

### Statistical Methods

Univariate analysis was performed for categorical variables using χ^2^ or Fisher’s exact test, as appropriate using SAS software, Version 9.4 (SAS Institute, Cary, NC).

Ethical approval was obtained at UCLA Fielding School of Public Health and the Kinshasa School of Public Health.

## RESULTS

### Study Population

Fourteen individuals were identified as survivors of the 1976 EBOV outbreak in Yambuku, DRC. Data on survivor characteristics are presented in Table 1. Eight participants were male and 6 were female, ranging from 55 to 86 years of age (between 15 and 46 years of age at the time of infection). Six of these individuals were classified as confirmed EBOV cases and the additional 8 were classified as suspected cases based on the DRC Ministry of Health records obtained from the original outbreak investigation of 1976–77 [2]. Ethical approval was obtained from all participating institutions and informed consent was obtained from all participants in local language.

**Table 1.** Demographic Information and Antibody Profile From 1976 EBOV Zaire Ebola Virus Disease Survivors, Yambuku, Democratic Republic of the Congo (n = 14)

Demographic Information					Serologic Results			Neutralization Assays (% Neutralization)		Plaque Reduction Neutralization Test (PRNT)^f^			
										No Complement		With Complement	
Ebola Survivor (ES)	Age at Infection	Current Age^a^	Sex	CaseClassification	GP^b^	NP^b^	VP40^c^	Pseudo- Virus^d^	Entry Inhibition^e^	PRNT (50%)	PRNT (80%)	PRNT (50%)	PRNT (80%)
1	26	66	M	Suspected	~ +	~ +	−	27.53%	32.28%	−	−	−	−
2	15	55	F	Suspected	~ +	~ +	−	32.66%	42.07%	−	−	−	−
3	15	55	M	Suspected	~ +	~ +	−	29.26%	36.03%	−	−	−	−
**4**	**28**	**68**	**M**	**Confirmed**	+	+	−	**70.14%**	**75.82%**	**1:30**	−	**1:30**	−
5	30	70	F	Confirmed	~ +	+	−	50.40%	49.91%	−	−	−	−
6	27	67	M	Confirmed	+	~ +	−	17.58%	23.79%	−	−	−	−
7	28	68	M	Suspected	−	~ +	−	13.13%	36.12%	−	−	−	−
**8**	**22**	**62**	**M**	**Confirmed**	+	+	+	**69.12%**	**80.10%**	**1:60**	**1:30**	**1:30**	−
9	28	68	F	Suspected	+	~ +	−	37.38%	37.89%	−	−	−	−
10	33	73	F	Suspected	−	~ +	−	41.33%	51.63%	−	−	−	−
11	21	61	M	Suspected	~ +	~ +	−	10.14%	35.82%	−	−	−	−
**12**	**46**	**86**	**F**	Suspected	~ +	−	−	**22.77%**	**17.98%**	**1:120**	−	−	−
**13**	**22**	**62**	**F**	**Confirmed**	~ +	+	+	**42.85%**	**39.40%**	**1:30**	−	−	−
14	25	65	M	Confirmed	+	+	+	41.75%	36.38%	−	−	−	−

^a^Current age at time of survey administration, January 2016.

^b^Patient immunoreactivity to Ebola virus Zaire glycoprotein (GP) and nucleoprotein (NP) recombinant viral proteins, assessed by ELISA. ~ +, indicats a likely reactive survivor.

^c^Patient immunoreactivity to Ebola virus Zaire viral protein 40 (VP40) antigen, assessed by a luciferase immunoprecipitation assay (LIPS).

^d^Results from pseudovirus neutralization assays are displayed as percent of neutralization as compared to controls.

^e^Entry inhibition using Ebola virus Zaire-like particles in a G-Luciferase (GLuc) complementation/neutralization assay, neutralization is displayed as a percent of infection compared to controls.

^f^Plaque reduction neutralization test (PRNT) using live Ebola virus Zaire under BSL-4 conditions. Displayed are the patient serum dilutions necessary to achieve 50% and 80% neutralization, with and without complement proteins present. All 14 subjects were run on PRNT platform and those that did not reach at least 50 or 80% plaque reduction are indicated by −, survivors harboring protective antibodies that were able to neutralize live EBOV to at least 50% of control are marked in bold font.

### EBOV Glycoprotein, Nucleoprotein, and Viral Matrix Protein 40 Immunoreactivity

**Figure 2.**
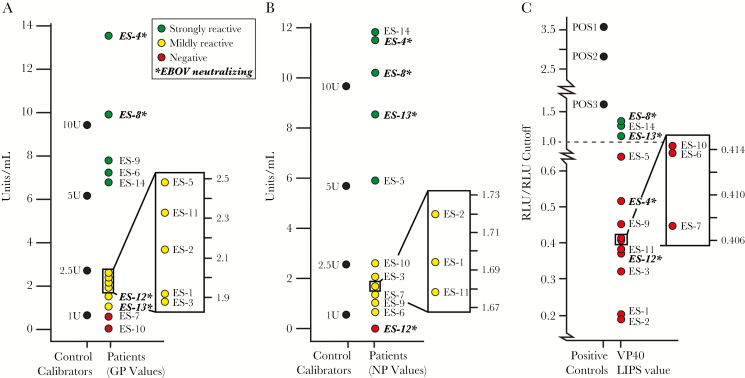
Immunoreactivity assay measures for the 14 remaining Ebola virus Zaire (EBOV) survivors from the 1976 Yambuku outbreak in the Democratic Republic of Congo (DRC). *A*, Antibody titer values measuring reactivity to EBOV glycoprotein (GP) using enzyme-linked immunosorbent assay (ELISA), with standard concentrations of purified IgG against EBOV GP used to generate standard curves. *B*, Antibody titers measuring reactivity to EBOV nucleoprotein (NP) using ELISA, with standard concentrations of purified IgG against EBOV NP used to generate standard curves. *C*, Relative luciferase signal obtained after immunoprecipitating EBOV viral protein 40 (VP40)-Luc using patient serum samples. The positive controls used for the luciferase immunoprecipitation system study were obtained from convalescent patient sera during the 2014 Boende EBOV outbreak in the DRC. Samples in bold indicate those that were determined to neutralize live EBOV using the downstream plaque reduction neutralization test (PRNT) to at least 50% of input control. Abbreviations: ES, Ebola survivor; RLU, relative light unit.

We first examined if the known Ebola survivors (ES) exhibited immunoreactivity to EBOV glycoprotein (GP), EBOV nucleoprotein (NP), and EBOV viral matrix protein 40 (VP40) 40 years after initial infections. Among the 14 survivors, 7 (ES-1, 2, 3, 5, 11, 12, and 13), exhibited mild to moderate reactivity to EBOV GP with titers >1 U/mL and 5 survivors (ES-4, 6, 8, 9, and 14) were considered strongly reactive with a titer >5 U/mL, as measured by the commercially available ADI GP ELISA using a serum dilution factor of 1:200 (Figure 2A). When assessing reactivity to NP, we identified 5 survivors (ES-4, 5, 8, 13, and 14) who exhibited strong immunoreactivity, with a titer above 5 U/mL and 8 survivors (ES-1, 2, 3, 6, 7, 9, 10, and 11), with mild to moderate responses (titers > 1 U/mL), as measured by the ADI NP ELISA using a serum dilution factor of 1:200(Figure 2B). There were far fewer survivors exhibiting reactivity against VP40, with only 3 individuals that were considered to be immunoreactive (Figure 2C).

### EBOV Pseudovirus Neutralization Assays

**Figure 3.**
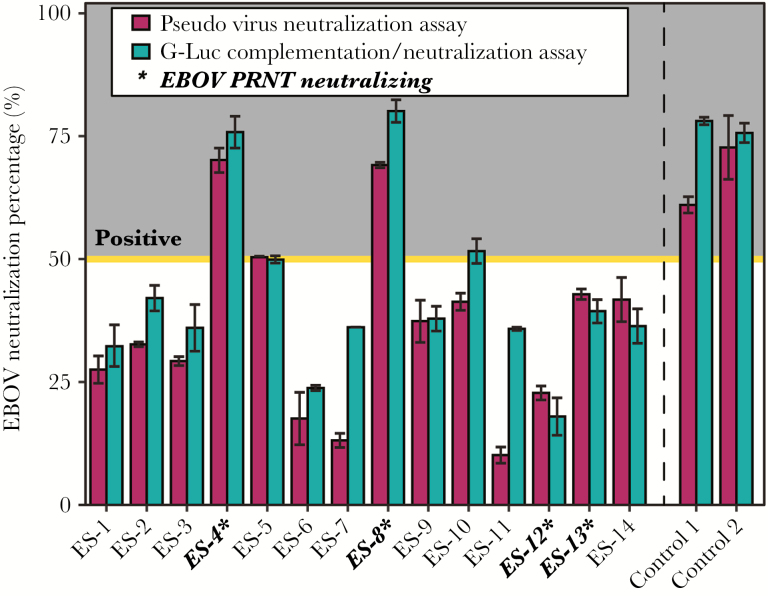
Ebola virus Zaire (EBOV) pseudotype neutralization. Representation of neutralization capacity in all 14 of the Yambuku survivor cohort using both the pseudovirus and the more sensitive G-Luciferase (GLuc) entry inhibition assay. Four of 14 Ebola survivors (ES) were able to reach 50% neutralization of control in at least of the 2 assays when normalized to negative control luciferase signals at a 1:50 serum dilution. Controls were obtained from convalescent patient sera during the 2014 Bonede EBOV outbreak in the Democratic Republic of the Congo. Those survivors highlighted in bold were successfully able to neutralize live EBOV using the downstream plaque reduction neutralization test (PRNT). Bars represent standard deviation between the duplicate tests run for each ES.

Once it was established that 100% of our study cohort were seropositive for EBOV GP, NP, or VP40, we next assessed if the reactive individuals were also capable of neutralizing virus. Three survivors (ES-4, 5, and 8) demonstrated capacity to neutralize the pseudovirus >50% using a serum dilution factor of 1:50. Using Ebola VLPs in a GLuc entry inhibition assay, which are thought to more closely mimic live EBOV in regards to eliciting immunological responses and protection [15, 23, 24], we found that 3 survivors (ES-4, 8, and 10) were capable of neutralization (>50%) and one (ES-5) demonstrated borderline capacity to neutralize in both assays (50.40% and 49.91%) again using a serum dilution factor of 1:50. Collectively, between these 2 assays, 4 survivors (29.0%) were identified as retaining neutralizing antibodies (Figure 3).

### Plaque Reduction Neutralization Test Using Live EBOV

**Figure 4.**
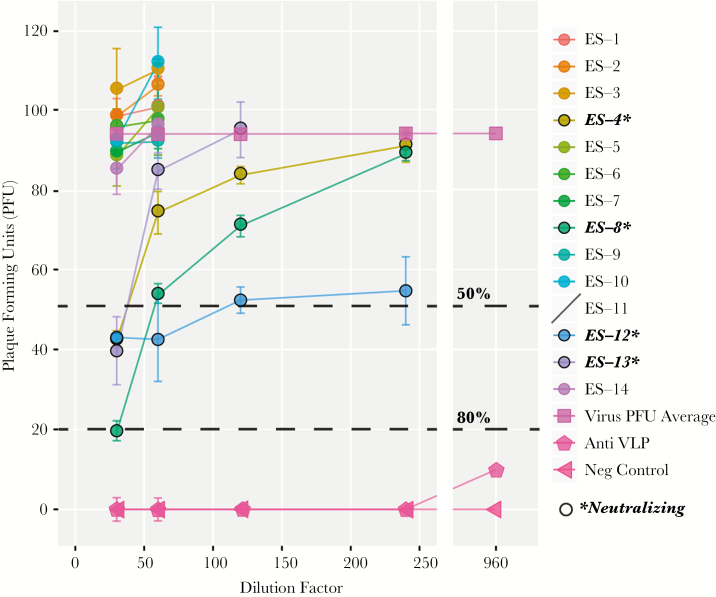
Plaque reduction neutralization test (PRNT) using live Ebola virus Zaire (EBOV). Using a PRNT assay with live EBOV, neutralization capacity was assessed in survivors from the 1976 Yambuku, Democratic Republic of the Congo Ebola outbreak. As highlighted, 4/14 of the Ebola survivors (ES) were able to neutralize at least 50% of live virus and 1 of the 4 neutralizing survivors had a strong enough response to neutralize 80% of virus on the PRNT. Bars represent the standard deviation obtained from the data between multiple tests conducted using serum from each ES. Abbreviation: VLP, virus-like particle.

After the establishment that a subset of survivors 40 years after initial infection showed neutralization capabilities using pseudo-type viral assays, we next sought to test neutralization capacity using live EBOV. Four survivors (ES-4, 8, 12, and 13) were able to neutralize live virus using a PRNT, 2 of which (ES-4 and 8) demonstrated strong neutralization using the pseudovirus assay, as well as strong reactivity in 2 or 3 anti-EBOV IgG assays, using recombinant viral proteins. Subjects ES-4 and 13 neutralized EBOV to at least 50% using a 1:30 dilution with complement proteins inactivated, while subject ES-8 showed even stronger trends, neutralizing 50% at 1:60 dilution and 80% at 1:30 dilution with complement proteins inactivated. Despite the inability to neutralize pseudovirus, our PRNT revealed that subject ES-12 neutralized live EBOV to at least 50% of control at a 1:120 serum dilution with nonactive compliment proteins. It should be noted, however, that the neutralization curve is atypical for ES-12, which may indicate that this patient simply exhibited nonspecific inhibition, or toxicity, as opposed to actually having specific EBOV neutralization capabilities (Figure 4).

## DISCUSSION

The recent EVD outbreak highlights the need to develop effective therapeutics or vaccines that limit pathogenesis and viral spread. Currently, there is a growing body of evidence that offers hope for finding ways to pharmacologically mimic or boost the natural resistance that some individuals appear to have towards EBOV infection. Viral proteins play a key role in host–virus interactions in EBOV infection [25] and the GP is the major determinant of entry and cellular tropism, an important protein involved in pathogenesis [14], and the primary target for protective immunity. However, in addition to GP, NP or other viral proteins could individually or synergistically constitute effective targets for future vaccine therapy [26]. Studies of EVD survivors may provide critical data to inform strategies for mimicking the resilience, which some individuals have demonstrated when infected with EBOV.

Here we describe the first documentation of live EBOV neutralizing antibody persistence within a subset of the oldest known EVD survivors, 40 years after acute EBOV infection. Additionally, these data represent the longest documented serological response to individual recombinant Ebola viral proteins (GP, NP, and VP40) and live EBOV. Until now, the longest duration of antibody responses to EBOV infection measured had been 11 years [9], thus our study provides evidence that antibody responses against EBOV can be long-lived and extend significantly further beyond known filovirus infections than previously documented. While the number of available survivors from this outbreak is limited, this is the only study to our knowledge that examines long-term immune response to EBOV in survivors on this time scale.

Despite the identifying levels of GP (12/14) and NP (13/14) reactivity in our study cohort (>1 U/mL), we only identified 3/14 survivors as retaining immunoreactivity to VP40. These findings are comparable to an earlier study of serological responses to the related Sudan Ebola virus (EBOV-S), suggesting that VP40 appears to be less commonly immunogenic than GP or NP [27]. As a gold standard, live EBOV neutralization was measured using a PRNT to determine if these EVD survivor samples could neutralize actual virus in vitro 40 years after infection. Four (29%) survivors had anti-Ebola neutralizing titers, 2 of which also demonstrated strong neutralization using the pseudovirus assay. As noted, the PRNT curve for ES-12 is atypical, raising the possibility that the mode of neutralization for this particular survivor may not be EBOV specific, but rather nonspecific inhibition. Of the 4 survivors with neutralizing antibodies, all reacted to viral protein GP, with survivors 4 and 8 being considered strongly reactive, and survivors 12 and 13 considered mildly reactive. Three survivors reacted strongly to NP (ES-4, 8, and 13), with one PRNT neutralizer showing no response to this recombinant protein. The 2 survivors (ES-8 and 13) with the highest neutralization capacity on the PRNT demonstrated reactivity to VP40.

Unlike other studies that have shown that human sera containing Mannose-binding lectin can enhance instances of neutralization of EBOV and MARV pseudovirus [28], here it appears that compliment did not have any significant effect on enhancing antibody-mediated EBOV neutralization capacity. More work will be necessary to accurately assess the best measures for predicting neutralization, as there is variability in neutralizer response to GP, NP, and VP40. However, as an initial screen, those who are NP and GP reactive appear more likely to neutralize the virus in downstream applications. Given that within this study cohort, the majority of survivors react at least mildly to GP, yet are unable to neutralize may indicate that GP response is not necessarily the best indicator of downstream neutralization. Additionally, 1 of the strongest PRNT neutralizers had extremely low response to GP, yet high responses to NP and VP40. However, it is also important to note that protective antibodies are not necessarily always neutralizing using in vitro assays [29], and leads to the possibility that in vivo, our survivor cohort may all be protected from reinfection, because there have not been documented cases of reinfection with EBOV.

Given the small sample size, we were not able to find any significant trends in symptoms reported, exposure routes, or other demographic factors that predicted neutralizing antibody response 40 years postinfection. However, these results agree with previous studies, which have found known survivors harboring detectable antibodies against viral proteins or full virus antigen [9, 10, 27]. Interestingly, survivor ES-8, whose sera demonstrated the greatest ability to neutralize live virus, was the only health-care worker enrolled in our study, indicating that these types of individuals should be investigated in greater depth during future research. Questionnaire data from this individual indicated multiple exposures to Ebola during the outbreak, with percutaneous exposure as well as contact with patient bodily fluids. The high levels of immunoreactivity seen in other individuals may also be the result of re-exposure through asymptomatic, endogenous, or sylvatic sources in an endemic region [25, 30, 31], although as mentioned, human reinfection with EBOV has not been documented. Another possibility is that related viruses may also stimulate EBOV humoral immunity, enabling this continued protective response observed in some survivors [27]. Alternatively, these individuals may have inherent genetic or regulatory differences that enable them to retain EBOV response and neutralization capabilities long after initial infection, whereas others lose this ability at a more rapid rate. Irrespective of mechanism, these survivors with specific, potent, and enduring immunological responses may guide beneficial research in preventing or treating EVD in inevitable future reoccurrences.

Participants in this study represent the long-term survivors of EVD from the 1976 Yambuku outbreak, those that survived long enough to be included in this study, which may not be a representative sample of survivors from this first documented outbreak. However, these individuals are the only known living survivors of the first recorded EBOV outbreak in history and represent a unique and highly important cohort, enabling the opportunity to glean insight into the long-term immune response against this potent pathogen. Life expectancy of this population is among the lowest in the world (58 for males, 62 for females) [32], and to find survivors 40 years after one of the deadliest Ebola outbreaks recorded presented numerous challenges, yet better than expected outcomes. This is an aging cohort and the aperture for studies of this nature is rapidly closing. Additional studies of the Yambuku survivors may provide an understanding of the health challenges that will face the survivors from the large West African Ebola outbreak.

This report documents anti-Ebola immunity to viral antigens 40 years after the initial infection, but, most interestingly, we demonstrate that a subset of these survivors are still capable of neutralizing live virus. The duration of the anti-Ebola humoral response fills a knowledge gap within the field and provides evidence that infection with EBOV can trigger a life-long humoral immune response, and in instances neutralize live virus in vitro.

## Notes

***Author contributions.*** A. W. R., M. S. B., N. H., R. D., P. M., V. H. A., C. M. R., and B. P. N. formulated project design, collected/analyzed data and contributed to manuscript preparation. K. L., I. S., L. K. Y., C. S., and G. S. generated, analyzed, and interpreted data generated under BSL-2 conditions and contributed to the final manuscript. G. O., J. P., J. L., R. S. B., P. J., and L. H. generated, analyzed, and interpreted data generated under BSL-4 conditions. D. L. H. provided manuscript assistance along with data that was gathered from the original 1976 study. B. K. I., E. O. W., and J. J. M. T. oversaw and contributed to ground efforts and data collection on site in DRC.

***Acknowledgments.*** Many individuals contributed to the design of this manuscript and data collection in the field. We thank the survivors of the Yambuku Ebola outbreak of 1976 and the community of Yambuku and Bumba Health Zones. In particular, we thank, Dr Alain Mbobo, Dr Alexis Mwanza, Dr Danny Bayekele, Landrine Bolela, Sukato Manzomba, Dr Ado Bwaka, Jose Ngamboli, Nathalie Kavira, Odile Falanka, John Manienga, Rachel Mutombe, and the DRC Ministry of Health for their assistance with field logistics, contacting survivors and the implementation of this study. We also thank and Allen Lipson for assistance in graphic preparation and Dr Joel Breman, Dr Karl Johnson, and Dr Peter Piot for their advice and assistance with locating historical records of the Yambuku outbreak and commentary on our manuscript. The pRen2 vector for expression of *Renilla* luciferase fusion proteins was contributed by Peter Burbelo, NCI.

***Financial support.*** This work was supported by the Bill and Melinda Gates Foundation Grant numbers: OPP1066684 (A. W. R.), OPP1151786 (A. W. R.), and 20153705 (via Atreca Inc.) (A. W. R.). The Faucett Catalyst Fund, NIAID Grant # R21AI107420 (G. S.), and DFG fellowship STE 2237/1-1 (G. S.). M. S. B. is supported by the Fogarty International Center of the National Institutes of Health (NIH) under Award Number D43TW009343 and the University of California Global Health Institute (UCGHI); The content is solely the responsibility of the authors and does not necessarily represent the official views of the NIH or UCGHI.

***Potential conflicts of interest.*** All authors: No reported conflicts of interest. All authors have submitted the ICMJE Form for Disclosure of Potential Conflicts of Interest. Conflicts that the editors consider relevant to the content of the manuscript have been disclosed.
